# *Zingiber officinale* and *Vernonia amygdalina* Infusions Improve Redox Status in Rat Brain

**DOI:** 10.1155/2022/9470178

**Published:** 2022-09-26

**Authors:** Damilare Emmanuel Rotimi, Goodnews Mavoghenegbero Ben-Goru, Ikponmwosa Owen Evbuomwan, Tobiloba Christiana Elebiyo, Mohammed Alorabi, Abdullah Farasani, Gaber El-Saber Batiha, Oluyomi Stephen Adeyemi

**Affiliations:** ^1^SDG 03 Group-Good Health & Well-Being, Landmark University, Omu-Aran 251101, Kwara State, Nigeria; ^2^Department of Biochemistry, Medicinal Biochemistry, Nanomedicine & Toxicology Laboratory, Landmark University, PMB 1001, Omu-Aran 251101, Nigeria; ^3^Department of Microbiology, Cellular Parasitology Unit, College of Pure and Applied Sciences, Landmark University, PMB 1001, Omu-Aran 251101, Nigeria; ^4^Department of Biotechnology, College of Sciences, Taif University, P.O. Box 11099, Taif 21944, Saudi Arabia; ^5^Department of Medical Laboratory Technology, Biomedical Research Unit, Medical Research Center, College of Applied Medical Sciences, Jazan University, Jazan 45142, Saudi Arabia; ^6^Department of Pharmacology and Therapeutics, Faculty of Veterinary Medicine, Damanhour University, Damanhour 22511, AlBeheira, Egypt; ^7^Laboratory of Sustainable Animal Environment, Graduate School of Agricultural Science, Tohoku University, 232-3 Yomogida, Naruko-Onsen, Osaki, Miyagi 989-6711, Sendai, Japan

## Abstract

The study investigated the effects of *Zingiber officinale* root and *Vernonia amygdalina* leaf on the brain redox status of Wistar rats. Twenty-four (24) rats weighing 160 ± 20 g were randomly assigned into four (4) groups, each with six (6) rats. Animals in Group 1 (control) were orally administered distilled water (1 mL), while the test groups were orally administered 5 mg/mL of either *Z. officinale, V. amygdalina* infusion, or a combination of both, respectively, for 7 days. The rats were sacrificed at the end of treatments and blood and tissue were harvested and prepared for biochemical assays. Results showed that administration of *V. amygdalina* and *Z. officinale,* as well as their coadministration, reduced the levels of malondialdehyde (MDA), nitric oxide (NO), acetylcholinesterase (AChE), and myeloperoxidase (MPO) in rat brain tissue compared with the control group. Conversely, coadministration of *V. amygdalina* and *Z. officinale* increased the levels of reduced glutathione (GSH) in rat brain tissue compared with the control group. However, the administration of the infusions singly, as well as the combination of both infusions, did not have any effect on the rat brain levels of glutathione peroxidase (GPx) and catalase (CAT) antioxidant enzymes compared to the control. Taken together, the findings indicate that the *V. amygdalina* and *Z. officinale* tea infusions have favorable antioxidant properties in the rat brain. The findings are confirmatory and contribute to deepening our understanding of the health-promoting effects of *V. amygdalina* and *Z. officinale* tea infusions.

## 1. Introduction

Over time, man has gradually gained knowledge and application of plants as sources of food and medicine through trial and error, and he has become able to source for his needs from his immediate environment. The application of these plants is being refined bit by bit due to technological and scientific advances. In many regions of the world, medicinal plants contribute to primary health care delivery especially in their use as vital sources of pharmaceutical and therapeutic products, with approximately 80% of the human population globally depending on natural products for primary health care [1, 2]. Medicinal plants are used singly or in combination with other substances (to increase treatment efficacy for several therapies) in the preparation of traditional or alternative medicines [3, 4].

Ginger (*Zingiber officinale),* belonging to the family Zingiberaceae, is a popular spice and herb used as a delicacy. *Z. officinale* has long been in use therapeutically and is currently validated as a potent medicinal spice to manage numerous diseases such as diabetes, hypertension, cancer, ulcer, diarrhea, cold, cough, spasms, and vomiting [5]. It is an effective spasmolytic, antipyretic, antiemetic, antioxidant, antiulcer, analgesic, hypotensive, antidiabetic, and anti-inflammatory agent containing scented essential oils and spicy oleoresins. The phenolic compounds (gingerols, shogaols, paradol, and gingerdiones) in *Z. officinale* have been discovered to be the source of their pharmacological benefits [5]. On the other hand, bitter leaf (*Vernonia amygdalina*) is a shrub or small tree belonging to the family Asteraceae that grows predominantly in tropical regions in Africa and Asia with the leaves having a characteristic bitter taste. For hundreds of years, bitter leaf serves as fodder, food, and herbal medicine [6]. *V amygdalina* is rich in minerals such as potassium, magnesium, zinc, iron, and vitamins A, C, and E [7]. The shrub has been known to exhibit profound pharmacological properties such as antimalarial, antioxidant, antimicrobial anticancer, neuroprotective, anti-inflammatory, and anthelminthic properties [8–12]. The pharmacological properties of the bitter leaf have been reported to be connected to its phytochemical constituents which include flavonoids, steroidal saponins, alkaloids, sesquiterpene lactones, anthraquinones, polyphenolics, and coumarins [3, 13]. Interestingly, *Z. officinale* and *V. amygdalina* are useful components in traditional medical systems like Ayurveda to manage neurodegenerative conditions [14, 15].

Oxidative phosphorylation leads to the production of oxidants and free radicals including reactive oxygen species (ROS) and reactive nitrogen species (RNS), the two most important signaling molecules that help to sustain cellular proliferation and differentiation, trigger stress-responsive survival mechanisms, and maintain cellular homeostasis [16–18]. Under normal physiological conditions at redox equilibrium, harmful effects of the production of ROS and other free radicals during aerobic metabolism are counterbalanced by the antioxidant system, and in this way, the organs in the body including the brain efficiently regulate their oxygen consumption and redox generation capacity [19, 20]. However, when the production of ROS, other free radicals, and radical reactive species surpasses the scavenging ability of the antioxidant response system, a situation referred to as redox imbalance ensues [21]. The redox imbalance could cause oxidative cellular damage because of lipid peroxidation as well as protein and nucleic acid oxidation. The resulting oxidative stress could mediate the pathogenesis and pathophysiology of several diseases including neurodegenerative disorders and cardiovascular diseases [22–26].

The brain is one of the most metabolically active organs in the body and its oxygen consumption and redox generation capacity is under strict control. Although the brain of an adult human being weighs about 1.4 kg, it consumes approximately 20% of the total basal oxygen to power its high metabolic activity [27]. In addition, the low endogenous antioxidant defense mechanism of the brain makes it highly sensitive to oxidative damage. Therefore, ischemic/hypoxic or hyperoxic condition raises the levels of free radicals and disrupts redox homeostasis in the brain which could lead to cellular death [28, 29]. Various types of antioxidants play significant roles in redox homeostasis. These antioxidants comprise vitamins A, C, and E; glutathione, coenzyme Q, bilirubin, and ferritin; and endogenous antioxidant enzymes such as glutathione peroxidase (GPx), catalase (CAT), superoxide dismutase (SOD), glutathione reductase (GR), and heme oxygenase (HO) [30, 31]. Furthermore, many medicinal plants and their products have been shown to possess antioxidant defense capacity that helps protect against oxidative damage and maintain redox homeostasis [32–34]. Therefore, the present study aimed to determine the effect of *Z. officinale* and *V. amygdalina* infusions on rat brain redox status.

## 2. Materials and Methods

### 2.1. Identification and Authentication of the Sample

*Z. officinale* roots were obtained from Omu-Aran market, Kwara state, while *V. amygdalina* leaves were acquired from Landmark University, Omu-Aran. Both plants were identified and authenticated at the Department of Plant Biology, University of Ilorin, Kwara State with voucher numbers; UILH|001|1023|2021 for *Z. officinale* and UILH|002|1083|2021 for *V. amygdalina*.

#### 2.1.1. Preparation of *Z. officinale* and *V. amygdalina* Infusion

The *Z. officinale* root was washed with water, peeled, sun-dried, and pulverized into powder. The *V. amygdalina* leaf was washed, sun-dried, and the stalk was removed from the leaf before it was pulverized into powder. To make the infusion, the plant powder in the tea bag was steeped in hot water (5 mg/mL, 100°C) for 3–5 minutes [35]. The infusion was allowed to cool at room temperature (25°C) before use.

### 2.2. Phytochemical Analysis

#### 2.2.1. Qualitative Phytochemical Screening

Qualitative screening for phytoconstituents of ginger and bitter leaf tea infusions was performed as previously described [13]. The phytochemicals assayed included saponin, phenolic compounds, water-soluble phenol, water-insoluble phenol, free and combined anthraquinones, flavonoids, poly steroid, cardiac glycosides, terpenoids, alkaloids, and tannins.

#### 2.2.2. Animal Grouping and Treatments

The animals (24 rats) were obtained from the Department of Biochemistry, University of Ilorin, Nigeria. They were housed in a clean cage, allowed to acclimatize for 14 days, and administered pelletized feed and water. The rats were assigned randomly into four (4) groups, consisting of six (6) rats each. Animals in Group 1 were orally administered distilled water (1 mL). Groups 2, 3, and 4 were orally administered 5 mg/mL of *Z. officinale, V. amygdalina*, and an oral combination of *Z. officinale* and *V. amygdalina* infusion, respectively. The treatments lasted for 7 days.

The animals received humane care in compliance with the institution's guidelines and criteria as outlined in the National Institute of Health (NIH) Guidelines for the care and use of laboratory animals, and this work had approval from the ethical committee of the Department of Biochemistry at Landmark University, reference number: LUAC/2021/003A.

After the last treatments, the animals were fasted overnight and sacrificed under mild anesthesia of diethyl ether. The brain was harvested, weighed, and homogenized in an isotonic medium (0.25 M sucrose solution). The homogenate was centrifuged at 5000 rpm for 10 min to obtain supernatants, which were used for biochemical analysis. A section of the brain was also preserved in 10% buffered neutral formalin (BNF) for a histopathology examination.

#### 2.2.3. Biochemical Assays

The brain homogenates collected were used in the biochemical determination of redox markers. Total protein content in the rat brain homogenate was determined according to Gornall et al. [36]. Superoxide dismutase (SOD) and glutathione peroxidase (GPx) activities were assayed as previously reported by Misra and Fridovich [37] and Rotruck et al. [38], respectively. The procedures described by Beers and Sizer [39] and Pulli et al. [40] were used to assay for catalase (CAT) and myeloperoxidase (MPO), respectively. For the determination of reduced glutathione (GSH) and malondialdehyde (MDA) levels, the methods described by Beutler and Yeh [41] and Varshney and Kale [42] were used, respectively. Nitric oxide (NO) level and acetylcholinesterase (AChE) activity were determined using Adeyemi et al. [43] and Ellman et al. [44], respectively. DNA fragmentation was carried out following the procedure described by Perandones et al. [45].

#### 2.2.4. Histopathology Examination

After the rat brain was excised from the animal, it was fixed in 10% BNF and subsequently processed for histopathology examinations as described by Adeyemi and Akanji [46]. Photomicrograph capture and scoring for morphological changes were done at the Pathology Unit, University of Ilorin Teaching Hospital, Ilorin, Nigeria.

#### 2.2.5. Statistical Evaluation

The data were analyzed on GraphPad Prism Software (San Diego, CA, USA), using one-way ANOVA. Data are presented as the mean value of six replicates plus or minus the standard error of the mean (SEM). At *p* value of 0.05, mean values were considered significant.

## 3. Results

### 3.1. Qualitative Phytochemical Analysis

The qualitative phytochemical screening revealed the presence of saponin, phenolics, flavonoids, alkaloids, and anthraquinones in the tea infusions of ginger and bitter leaf. However, tannins were not detected in ginger infusion but were present in bitter leaf (Table 1).

**Figure 1:**
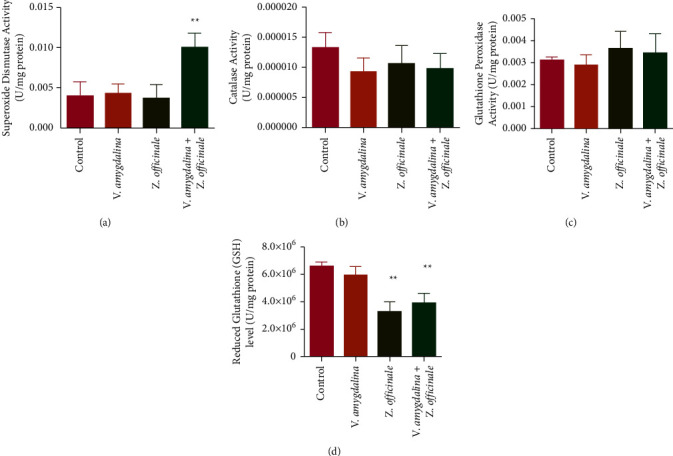
The effect of *Zingiber officinale* and *Vernonia amygdalina* on the levels of rat brain antioxidant molecules: (a) superoxide dismutase, (b) Catalase, (c) glutathione peroxidase, and (d) reduced glutathione. Each value is a mean of six replicates ± SEM. Values bearing the asterisk are significantly different compared to the control at *p* *<* 0.05.

The administration of Z. officinale and V. amygdalina infusions separately had no significant effect on the rat brain superoxide dismutase (SOD) activity relative to control. In contrast, the coadministration of both infusions significantly increased the rat brain SOD activity in comparison with control (Figure 1(a)). As shown in Figure 1(d), the separate administration of *Z. officinale* and *V. amygdalina* infusions as well as the coadministration of both infusions did not significantly change the rat brain catalase (CAT) activity in relation to control (Figure 1(b)). As represented in Figure 1(c), there were no significant changes in the rat brain glutathione peroxidase activity in relation to control when *Z. officinale* and *V. amygdalina* infusions were administered separately. In addition, the coadministration of both infusions had a negligible effect on the rat brain glutathione peroxidase activity in relation to control. Glutathione level in the rat brain was reduced when *Z.* officinale infusion only was administered, as well as the coadministration of both infusions, when compared with the control (Figure 1(d)).

**Figure 2:**
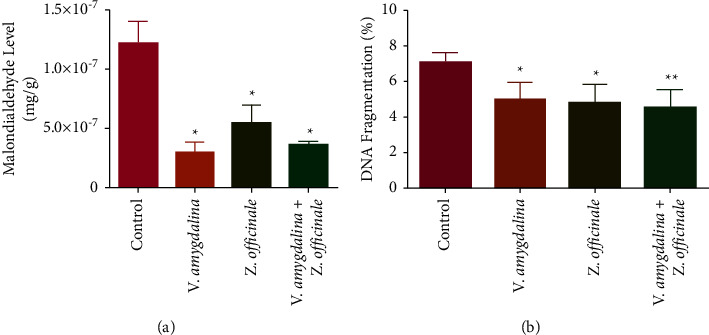
The effect of *Zingiber officinale* and *Vernonia amygdalina* on the level of rat brain oxidative stress markers: (a) malondialdehyde and (b) DNA fragmentation. Values are the mean of six replicates ± SEM. Bars with the asterisk are significantly different from the control at *p* < 0.05.

An appreciable decrease in the rat brain MDA level in comparison with the control was observed when the infusions were administered separately as well as coadministration of the infusions together (Figure 2(a)). In the same vein, the administration of the tea infusions separately or as a combination significantly decreased the level of the rat brain DNA fragmentation when compared with the control (Figure 2(b)).

**Figure 3:**
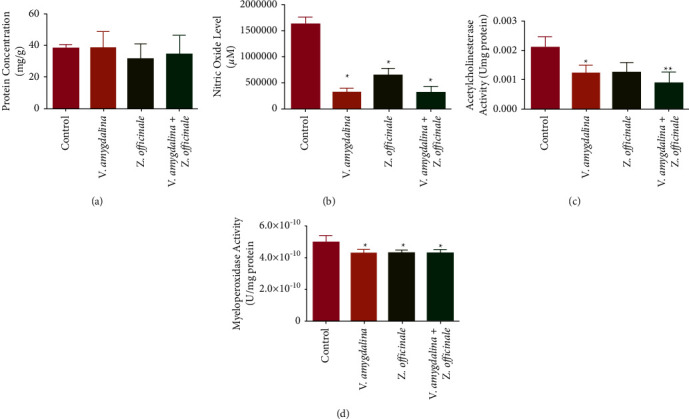
The effect of *Zingiber officinale* and *Vernonia amygdalina* on the level of rat brain biochemical parameters: (a) protein concentration, (b) nitric oxide, (c) acetylcholine esterase, and (d) myeloperoxidase. Values are the mean of six replicates ± SEM. Bars with the asterisk are significantly different from the control at *p* < 0.05.

Furthermore, the administration of the infusions separately, as well as in a combination, did not significantly affect the rat brain protein concentration when compared to control (Figure 3(a)). However, the administration of the infusions separately or as a combination significantly decreased the level of the rat brain nitric oxide (NO) in relation to control (Figure 3(b)).  Figure 3(c) shows a substantial decrease in rat brain AChE activity in comparison with the control when the infusions were administered singly or in combination. Similarly, the rat brain MPO activity decreased when the infusions were administered separately or as a combination (Figure 3(d)).

**Figure 4:**
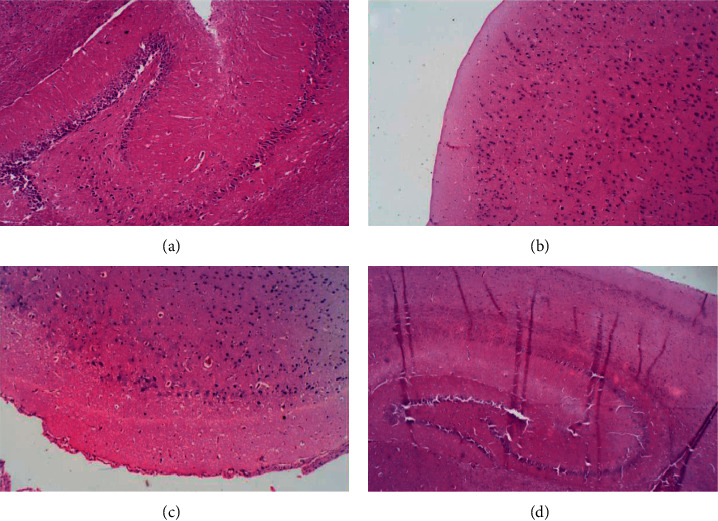
The effect of *Zingiber officinale* and *Vernonia amygdalina* on rat brain morphology: (a) control, (b) *Vernonia amygdalina,* (c) *Zingiber officinale,* and (d) combination of *Zingiber officinale* and *Vernonia amygdalina*. H&E staining (x100). The sections show normal brain tissue composed of preserved neuronal bodies surrounded by a fibrillary glial matrix (cerebral and hippocampal).

For all the treatment groups, the histopathology examination revealed normal brain tissue composed of preserved neuronal bodies surrounded by a fibrillary glial matrix (cerebral and hippocampal). No observation of degenerative changes or significant inflammation (Figure 4).

## 4. Discussion

This work investigated the effect of *Z. officinale* root and *V. amygdalina* leaf infusion on the redox status of the brain of rats to provide more understanding of the health-promoting effects of these commonly consumed medicinal plants. The phytochemical screening showed the presence of saponin, phenolics, flavonoids, free anthraquinones, and alkaloids. However, tannins were not detected in ginger but only in the tea infusions of bitter leaf and that of the combination of bitter leaf and ginger.

We found that the combined mixture of *V. amygdalina* and *Z. officinale* infusions led to a significant increase in SOD activity and an insignificant increase in GPx activity across all experimental groups when compared with the control. This finding could mean that the infusions enhanced the rat brain's oxidative defense status by improving the activities of SOD [47]. In addition, our study revealed that the combined administration of the infusions caused an insignificant increase in CAT activity but a significant decrease in reduced glutathione (GSH). GSH is an antioxidant molecule that quenches O_2_^•−^, HO^•^, and peroxynitrite anions (ONOO-) by the action of its thiol group, which donates a reducing equivalent to unstable radicals and anions that may have arisen due to respiratory processes and other metabolic activities in the body cells. In contrast to the increased antioxidant activity of SOD, CAT, and GPx, there was a significant decrease in the level of malondialdehyde (MDA), an end-product of lipid peroxidation during oxidative degradation of lipids usually caused by free radicals [48]. The coordinated function of enzymatic antioxidant systems including CAT, SOD, and GPx, and nonenzymatic antioxidants such as glutathione (GSH) and vitamin E play a significant role in the prevention of oxidative damage by ROS such as O_2_^•−^, HO^•^ and H_2_O_2_ [49, 50]. A significant decrease in the level of nitric oxide (NO), a proinflammatory agent, may indicate the anti-inflammatory action of the tea infusions. Nitric oxide (NO) participates in several physiological processes and is considered a free radical as it could accumulate and react with superoxide anion to form poisonous nitrite anion, thereby leading to nitrosative stress [51]. The decrease in the levels of both MDA and NO across all experimental groups in comparison with the control may be due to the antioxidant properties of *V. amygdalina* and *Z. officinale*. This implies that the tea infusions can suppress oxidative stress by inhibiting the production of MDA and NO in support of earlier reports [14, 52–54]. The tea infusions can limit the production of MDA and NO, and thereby prevent oxidative stress probably by enhancing the activities of the inherent enzymatic antioxidants in the rat brain. Myeloperoxidase (MPO) is a heme-containing peroxidase mainly expressed in neutrophils and, to a lesser extent, in monocytes. In the presence of H_2_O_2_ and halides (HOCl), MPO catalyzes the formation of reactive oxygen intermediates such as hypochlorous acid. Microbial death by neutrophils relies heavily on the MPO/HOCl system. In addition, MPO acts as a local mediator of tissue injury through inflammation [55]. The observed decrease in MPO activity due to the separate or combined administration of the tea infusions is consistent with the findings of Hussein et al. [56] and Erukainure et al. [57]. A decrease in MPO activity impedes neutrophil infiltration [58], supporting the anti-inflammatory effect of tea infusions. Furthermore, results showed a decrease in DNA fragmentation following the separate or combined administration of the tea infusions, corroborating an earlier report [57]. This fact further reinforces not only the antioxidant but also the anti-inflammatory potential of the tea infusions of *Z. officinale* and *V. amygdalina* and their capacity to protect against oxidative-induced DNA damage.

Acetylcholinesterase (AChE) is a serine hydrolase primarily responsible for the termination of signal transmission in the cholinergic system. Its substrate, acetylcholine (ACh), is a cholinergic neurotransmitter that has a strong effect on motor neurons involved in memory formation [59]. Therefore, the observed decrease in the activity of this AChE after a separate and combined administration of the tea infusions is similar to the findings reported in earlier studies [11, 59–61]. The present finding suggests that the tea infusions possess bioactive compounds that could decrease neurodegeneration or treat neurodegenerative diseases by modulating the activity of AChE. Protein is an important structural component of cells as well as a source of energy and one of the building blocks of human tissues. Extracellular and intracellular enzymes, as well as other proteins, contribute to the total protein concentrations [62]. The separate and the combined administration of the tea infusions of *V. amygdalina* and *Z. officinale* caused a negligible rise in total protein concentration, attributable to their capacity to boost the production of antioxidant proteins as reflected in the elevated levels of SOD and CAT. Furthermore, morphological examination of the rat brain revealed normal histological architectures of the cerebral and hippocampal in all the treatment groups. There are no features of degenerative changes or inflammation, thus corroborating the biochemical data and further underscoring the medicinal value of *V. amygdalina* and *Z. officinale* tea infusions in rat brains.

## 5. Conclusions

The data from this work showed that *V. amygdalina* and *Z. officinale* infusions improved brain antioxidants and reduced oxidative stress after a 7-day separate or combined oral administration. The results support the efficacy of the tea infusions in maintaining redox status/homeostasis in rat brains. Taken together, the findings support the health-promoting effects of *V. amygdalina* and *Z. officinale*. Future studies may focus on the long-term effects of the tea infusions separately or as a combination to gain a better understanding of the therapeutic benefits of the medicinal plants.

## Figures and Tables

**Table 1:** Qualitative Phytochemical tests of the infusion of ginger, bitter leaf, and a combination of Ginger + Bitterleaf.

Phytochemical compounds	Ginger	Bitter leaf	Ginger + bitter leaf
Saponin	**+**	**+**	**+**
Phenolic compounds	**+**	**+**	**+**
Water insoluble phenol	**+**	**+**	**+**
Flavonoids	**+**	**+**	**+**
Free anthraquinones	**+**	**+**	**+**
Tannins	-	**+**	**+**
Alkaloids	**+**	**+**	**+**

+: Detected -: Not detected.

## Data Availability

The raw data are available from the corresponding author upon request.

## Additional Points

*Institutional Review Board Statement*: The protocol used in handling the animals was approved by the ethical committee of the Department of Biochemistry at Landmark University and assigned the reference number: LUAC/2021/003A.
